# A case report of systemic embolic events associated with atrial fibrillation

**DOI:** 10.1002/ams2.235

**Published:** 2016-08-25

**Authors:** Hidehiko Nakano, Hiroshi Yamagami, Hisashi Ofuchi

**Affiliations:** ^1^ Emergency Department Shonan Kamakura General Hospital Kamakura Kanagawa Japan

**Keywords:** Atrial fibrillation, circulation, coagulopathy

## Abstract

**Case:**

An 82‐year‐old woman who had atrial fibrillation was found unconscious and was brought to the emergency department by ambulance. Her Glasgow Coma Scale score was 3, and an electrocardiogram showed ST segment elevation in V3 and V4. Cardiac ultrasonography showed left ventricular asynergy in the anterior wall, septum, and apex. Although dissection of the aorta was suspected, contrast computed tomography showed multiple arterial thromboses, including bilateral common carotid arteries and poor contrast in the left ventricle. Diffusion‐weighted images of magnetic resonance imaging showed a diffuse high‐intensity area in both cerebral cortices.

**Outcome:**

The diagnosis was multiple arterial thromboembolisms associated with atrial fibrillation. There was no available treatment because of massive multiple lesions and the patient died within 24 h of presentation.

**Conclusion:**

Extracranial systemic embolic events other than cerebral embolism could be critical complications associated with atrial fibrillation.

## Introduction

Cerebral embolism is a major complication of atrial fibrillation. However, systemic embolic events can also occur, and these could be a critical complication.

## Case

The patient was an 82‐year‐old woman who had a history of non‐ruptured brain aneurysm, mitral valve regurgitation, which was replaced with a biological valve, atrial fibrillation (Af), and right cerebellar infarction. Her son found her lying unconscious by her bed in the morning and called an ambulance. Her Glasgow Coma Scale score was 3 on arrival at the emergency department of our hospital. Her vital signs showed a blood pressure of 139/85 mmHg, heart rate of 100 b.p.m., body temperature of 36.4°C, and respiratory rate of 30 breaths/min. Her oxygen saturation was 85% on receiving 10 L/min and assisted ventilation with a bag valve mask. Her pupils were 2 mm in diameter, equally rounded, and reactive to light bilaterally. There was a subcutaneous hemorrhage in her forehead. Breathing sounds were clear bilaterally and systolic murmur was heard at the apex. Even though her right arm was warm, her left arm and both feet were cold. Pulses of the right carotid artery and right radial artery were normal, but pulses of the left radial artery and bilateral femoral arteries were weak. Muscle tonus was weak in all of the extremities. The patient's prescribed medications included warfarin 2.5 mg, aspirin 100 mg, spironolactone 25 mg, tolvaptan 7.5 mg, furosemide 80 mg, rabeprazole 10 mg, and iron sulfate 105 mg.

**Figure 1:**
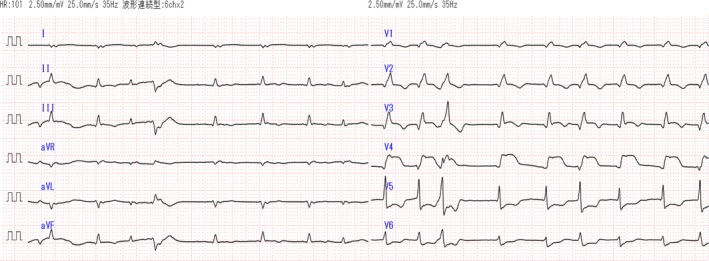
Electrocardiogram of an 82‐year‐old woman with atrial fibrillation on arrival at hospital.

An electrocardiogram carried out on arrival showed Af, an inverted T wave in V1 to V3, an abnormal Q wave in V1 to V4, and ST elevation in V3 and V4 (Fig. 1). A chest X‐ray showed prominent cardiomegaly, pulmonary congestion, and dilation of the upper mediastinum. Cardiac ultrasound, performed by an ultrasound technician, showed decreased wall contraction in the anterior wall, septum, and apex. Color Doppler ultrasound imaging did not detect blood flow in the abdominal aorta.

Laboratory data showed the following: creatine kinase (CK), 133 IU/L; CK‐MB, 17 IU/L; aspartate aminotransferase, 37 IU/L; alanine aminotransferase, 19 IU/L; blood urea nitrogen, 37.3 mg/dL; creatinine, 0.93 mg/dL; sodium, 141 mEq/L; potassium, 5.1 mEq/L; chloride, 109 mEq/L; glucose, 145 mg/dL; troponin I, 0.65 ng/mL; brain natriuretic peptide, 291.8 pg/mL; and prothrombin international normalized ratio, 1.29.

**Figure 2:**
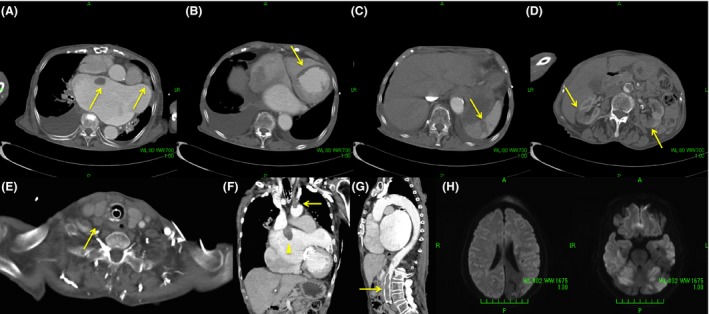
Contrast computed tomography scan and diffusion‐weighted image of magnetic resonance imaging in an 82‐year‐old woman with atrial fibrillation. A, Thrombi in the left atrium (arrows). B, Poor contrast in the left ventricle (arrow). C, Poor contrast in the spleen (arrow). D, Poor contrast in bilateral kidneys (arrows). E, Contrast defect in the right common carotid artery (arrow). F, Thrombus at the bifurcations of the left common carotid artery and subclavian artery (arrow) and thrombus in the left atrium (arrow head). G, Occlusion of the aorta (arrow). H, Diffuse high‐intensity area in the cerebrum and cerebellum.

We undertook head computed tomography (CT) to rule out intracranial hemorrhage, but there were no abnormalities, besides a previous right cerebellar infarction. Therefore, we performed contrast CT to examine dissection of the aorta. Contrast CT did not show dissection of the aorta, but we found emboli in bilateral common carotid arteries, the left subclavian artery, the celiac artery, and the distal abdominal aorta. Poor contrast in the left ventricle, spleen, and both kidneys was also observed. Some clots were recognized in the left atrium (Fig. 2). Brain magnetic resonance imaging was carried out and showed a diffuse high‐intensity area (Fig. 2). This finding suggested acute ischemic stroke in almost all of the patient's brain.

The patient's prognosis was so poor that cerebrovascular catheter intervention or any other embolectomy could not be undertaken. Intravenous heparin was given, but she died within 24 h.

## Discussion

Stroke is a well‐known complication of Af, but extracranial systemic embolic events (SEEs) can also occur. Systemic embolic events remain poorly defined. Wobo *et al*.^1^ first reported an analysis of SEEs based on a database of four large, randomized, clinical trials (ACTIVE‐A, ACTIVE‐W, AVERROES, and RE‐LY). In this analysis, the incidence of SEEs was 11.5% of clinically recognized thromboembolic events in patients with Af, with an anatomical distribution of 58% in the lower extremities, 10% in the upper extremities, 22% in the mesentery, 6% in the kidneys, 3% in the spleen, and 1% in other areas. Morbidity and mortality of SEEs are variable depending on the anatomical site.^1^ The hazard ratio for long‐term mortality of patients with visceral or mesenteric SEEs versus no events is 17.64, and that of patients with both SEEs and stroke is 23.82.^1^ Severe visceral SEEs and stroke occurred simultaneously in our case. The findings in our patient showed how severe SEEs may become.

We found that diagnosing SEEs from the beginning of the assessment of our patient was difficult because coma, acute coronary syndrome, and a weak pulse in the extremities mimic aortic dissection. We accidentally diagnosed SEEs after a contrast CT scan when we aimed to exclude acute aortic dissection. However, retrospectively, the following findings might be helpful for differentiating SEEs other than acute aortic dissection.

1. Vascular insufficiency was found only in the left arm and not in the right arm, which suggested that blood flow of the right brachiocephalic artery was spared. If acute aortic dissection was the cause of stroke, blood flow in the right internal carotid artery should be spared. However, neurological findings were coma and quadriplegia instead of right hemiplegia.
2. There was no pericardial effusion, dilatation of the ascending aorta, or a flap in the aortic arch in ultrasound.
3. There were no transient neurological symptoms in our patient, which occur in half of patients with ischemic brain stroke with acute aortic dissection.^2^

Systemic embolic events show various symptoms. Physicians should consider SEEs as a differential diagnosis when assessing patients with a history of Af.

Coronary thromboembolism is a rare complication of Af. We could not perform a coronary angiogram in this patient, but coronary embolism was suspected by the criteria proposed by Shibata *et al*.^3^ (Table 1).

**Table 1:** Proposed National Cerebral and Cardiovascular Center criteria for the clinical diagnosis of coronary artery embolism (CE)

Major criteria
Angiographic evidence of CE and thrombosis without atherosclerotic components
Concomitant coronary artery embolization at multiple sites^a^
Concomitant systemic embolization without left ventricular thrombus attributable to acute myocardial infarction
Minor criteria
<25% stenosis on coronary angiography, except for the culprit lesion
Evidence of an embolic source based on transthoracic echocardiography transesophageal echocardiography, computed tomography, or magnetic resonance imaging
Presence of embolic risk factors: atrial fibrillation, cardiomyopathy, rheumatic valve disease, prosthetic heart valve, patent foramen ovale, atrial septal defect, history of cardiac surgery, infective endocarditis, or hypercoagulable state
Definite CE
Two or more major criteria, or
One major criterion plus ≥ 2 minor criterion, or
Three minor criteria
Probable CE
One major criterion plus 1 minor criterion, or
Two minor criteria
A diagnosis of CE should not be made if there is
Pathological evidence of atherosclerotic thrombus
History of coronary revascularization
Coronary artery ectasia
Plaque disruption or erosion detected by intravascular ultrasound or optic coherence tomography in the proximal part of the culprit lesion

The present proposed diagnostic criteria for CE include three major and three minor criteria. Weighted scoring of the criteria is used to differentiate between definite and probable CE in patients with acute myocardial infarction.

a Multiple vessels within one coronary artery territory or multiple vessels in the coronary tree.

This case met one major criterion and two minor criteria.^3^ However, exclusion criteria of pathological evidence of atherosclerotic thrombus and plaque erosion detected by intravascular ultrasound and optic coherence tomography in the proximal part of the culprit lesion^3^ were not investigated in our patient. This is because the patient's prognosis was too poor to undertake coronary angiography and an autopsy was not performed.

There are some published case reports of acute coronary syndrome secondary to thromboembolism. Atrial fibrillation is the most frequent thromboembolic risk factor.^3^, ^4^ Coronary thrombosis most frequently occurs in the left anterior descending artery (LAD), followed by the right coronary artery, and occurs less frequently in the left circumflex artery.^5^, ^6^ Findings from an electrocardiogram of ST elevation in V3 and V4, laboratory data of positive CK‐MB and troponin I levels, a contrast CT scan with poor contrast in the anterior wall and septum of the left ventricle, and cardiac ultrasound of left ventricular wall motion asynergy in the anterior wall and septum suggested acute coronary syndrome of the LAD lesion in our patient. We could not definitely diagnose coronary embolism in this case because of a lack of coronary angiographic or autopsy information. However, acute coronary syndrome in our patient simultaneously occurred with SEEs. Therefore, coronary embolism in the LAD is also a possible diagnosis.

## Conclusion

Extracranial SEEs other than cerebral embolism could be critical complications associated with Af.

## Conflict of Interest

None.
